# scAMAC: self-supervised clustering of scRNA-seq data based on adaptive multi-scale autoencoder

**DOI:** 10.1093/bib/bbae068

**Published:** 2024-02-29

**Authors:** Dayu Tan, Cheng Yang, Jing Wang, Yansen Su, Chunhou Zheng

**Affiliations:** Key Laboratory of Intelligent Computing and Signal Processing of Ministry of Education, School of Artificial Intelligence, Anhui University, 230601 Hefei, China; Key Laboratory of Intelligent Computing and Signal Processing of Ministry of Education, School of Artificial Intelligence, Anhui University, 230601 Hefei, China; Key Laboratory of Intelligent Computing and Signal Processing of Ministry of Education, School of Artificial Intelligence, Anhui University, 230601 Hefei, China; Key Laboratory of Intelligent Computing and Signal Processing of Ministry of Education, School of Artificial Intelligence, Anhui University, 230601 Hefei, China; Key Laboratory of Intelligent Computing and Signal Processing of Ministry of Education, School of Artificial Intelligence, Anhui University, 230601 Hefei, China

**Keywords:** self-supervised clustering, multi-scale autoencoder, single-cell sequencing, attention mechanism, fuzzy clustering

## Abstract

Cluster assignment is vital to analyzing single-cell RNA sequencing (scRNA-seq) data to understand high-level biological processes. Deep learning-based clustering methods have recently been widely used in scRNA-seq data analysis. However, existing deep models often overlook the interconnections and interactions among network layers, leading to the loss of structural information within the network layers. Herein, we develop a new self-supervised clustering method based on an adaptive multi-scale autoencoder, called scAMAC. The self-supervised clustering network utilizes the Multi-Scale Attention mechanism to fuse the feature information from the encoder, hidden and decoder layers of the multi-scale autoencoder, which enables the exploration of cellular correlations within the same scale and captures deep features across different scales. The self-supervised clustering network calculates the membership matrix using the fused latent features and optimizes the clustering network based on the membership matrix. scAMAC employs an adaptive feedback mechanism to supervise the parameter updates of the multi-scale autoencoder, obtaining a more effective representation of cell features. scAMAC not only enables cell clustering but also performs data reconstruction through the decoding layer. Through extensive experiments, we demonstrate that scAMAC is superior to several advanced clustering and imputation methods in both data clustering and reconstruction. In addition, scAMAC is beneficial for downstream analysis, such as cell trajectory inference. Our scAMAC model codes are freely available at https://github.com/yancy2024/scAMAC.

## INTRODUCTION

Single-cell RNA sequencing (scRNA-seq) becomes an essential tool for studying cell heterogeneity and developmental processes [1]. It enables the measurement of gene expression in individual cells, considering the variations in gene activity and cellular characteristics. Compared to bulk RNA sequencing, the scRNA-seq technique has higher resolution and sensitivity, which describes the cell states of different cell types and subtypes in tissues and organs [2]. The rapid development of the technique has revolutionized transcriptomic studies and has provided deeper insights into biological processes that were previously inaccessible. Cluster assignment is an important step in the analysis of scRNA-seq data, which is able to identify different cell types and subtypes, and facilitate downstream analysis of scRNA-seq data. However, the high noise and sparsity of scRNA-seq data lead to a large number of zero values in their gene expression profiles [3, 4]. Potential technical biases during the amplification stage of scRNA-seq also affect the accuracy of gene expression values [5]. These erroneous gene expression values directly affect the cell clustering process, hindering downstream analysis.

Several clustering methods, including those that enhance K-means clustering, hierarchical clustering and graph-based clustering approaches, have been introduced to address challenges in scRNA-seq data analysis. K-branches [6] is a clustering method similar to K-means, which uses locally fitted half-lines to represent branches in cell differentiation trajectories and assigns cells to the nearest half-lines. Some methods are based on hierarchical clustering. CIDR [7] interpolates missing values to address ‘dropout events, and employs hierarchical clustering on the first few principal coordinates in scRNA-seq data. Seurat [8] constructs a shared nearest neighbor graph and uses a graph-based community detection algorithm called Louvain for clustering. However, traditional clustering methods often have difficulty detecting rare cell communities due to the sparsity and high noise of scRNA-seq data. Therefore, some methods consider using the idea of consensus clustering to overcome these problems. For example, SIMLR [9] uses multiple kernel functions to learn similarity measures between cells and improve clustering performance. SC3 [10] learns cell features from different perspectives using Euclidean distance, Pearson correlation and Spearman correlation, then obtains the final result using a consensus clustering method. SAME [11] obtains clustering solutions from multiple methods using a mixture model and selects the subset with the highest diversity to produce an improved ensemble solution. While these ensemble learning-based methods somewhat mitigate the impact of data noise on clustering results, they cannot effectively extract latent information in scRNA-seq datasets, and the computational cost of such algorithms increases dramatically with the size of the dataset.

Deep neural networks have demonstrated excellent performance in large-scale deep feature extraction in recent years due to their hierarchical structure and non-linear mapping ability. Therefore, deep learning-based clustering methods, broadly categorized into those based on autoencoders, graph neural networks and contrastive learning, have been widely applied in scRNA-seq data analysis. scDeepCluster [12] proposes a deep embedded clustering algorithm based on autoencoders, which combines the ZINB model [13] with deep embedded clustering [14] to optimize latent feature learning and clustering simultaneously. scGMAI [15] is a Gaussian mixture model based on autoencoders and FastICA. It uses autoencoders to reconstruct data, employs FastICA to reduce the dimensionality of reconstructed data and ultimately employs a Gaussian mixture model for clustering. Although scDeepCluster and scGMAI can latent features, they ignore the relationships between cells, which may lead to less accurate learned features. GraphSCC [16] establishes cellular structural relationships through graph convolutional networks (GCN) [17] and iteratively optimized low-dimensional representations and clustering objective functions using a dual self-supervised module. scGAC [18] designs a graph attention structure that captures cellular relationship through graph attention autoencoders. Attention mechanisms help scGAC assign different weights to different neighbors when propagating information in the neighborhood. scDSC [19] integrates a ZINB model-based autoencoder, a graph neural network module, and a mutual-supervised strategy to effectively handle noise, high dimensionality, and dropout events in scRNA-seq data. Although these GCN-based methods can learn cellular relationships, the accuracy of the constructed graph affects clustering performance. Contrastive-sc [20] proposes a self-supervised contrastive learning method for scRNA-seq data, which includes the representation learning stage and the clustering stage. scNAME [21] introduces a unique combination of mask estimation, contrastive learning with a global memory bank, contributing to accurate and robust clustering. However, these methods don’t fully exploit the latent feature information of cells, and their representation enhancement methods by masking specific parts of the input and contrastive loss may lead to false clustering results.

Several scholars have developed various data reconstruction methods to overcome the negative impact of ‘dropout events on downstream analysis of scRNA-seq data. DCA [22] achieves zero-value imputation through a redefined reconstruction loss. AutoImpute [23] learns the data distribution by training an autoencoder network to reconstruct the underlying true gene expression matrix. AutoClass [24] effectively filters out noise and recovers gene expression by adding a classifier branch to the autoencoder. scIGANs [25] uses a generative adversarial network to simulate real gene expression values and correct erroneous data. scGNN [26] is a method that utilizes a graph convolutional neural network (GCN) to construct a graph network representing cell relationships. Through training, it acquires low-dimensional features which are applied for clustering the data. The imputation-focused methods are not designed with modules specifically for clustering tasks, and there is no consensus on their effectiveness regarding data reconstruction quality. Furthermore, these deep models based on scRNA-seq data often ignore the interconnections and mutual influences between network layers. Many autoencoder-based methods do not fully utilize the information of the decoding layer and only focus on the features of the hidden layer. However, a single hidden layer feature cannot fully represent the deep relationships between cells.

Therefore, we propose a new self-supervised clustering method (scAMAC) based on an adaptive multi-scale autoencoder. The advantage of self-supervised learning lies in its ability to fully leverage the inherent structure of the data, achieved through cleverly designed tasks that enable the model to learn rich feature representations [27–29]. Inspired by the Efficient Paired-Attention [30] mechanism and the Efficient Channel Attention [31] mechanism, scAMAC utilizes the Multi-Scale Attention (MSA) mechanism to fuse the feature information from the encoder, hidden and decoder layers of the multi-scale autoencoder. It enables a comprehensive analysis of cellular characteristics at various resolutions, unveiling intra-scale cellular correlations and deep features that span multiple scales. The self-supervised clustering network calculates the membership matrix of the fuzzy k-means (FKM) algorithm using the fused latent features and optimizes the self-supervised clustering network based on the membership matrix. The adaptive feedback mechanism employed in scAMAC facilitates self-supervised learning and continuous optimization of model parameters, obtaining a more effective representation of cell features. During the operation, scAMAC not only achieves cell clustering but also data reconstruction through the decoding layer of the model. We compare scAMAC with seven advanced clustering methods and three deep learning-based imputation methods to demonstrate the superiority of scAMAC in scRNA-seq data clustering and reconstruction. Furthermore, we demonstrate clustering and cell trajectory construction through visualization.

## MATERIALS AND METHODS

### Data preprocessing

We conduct relevant experimental comparisons using highly competitive scRNA-seq datasets to demonstrate the effectiveness and potential value of the proposed method. We collect 14 commonly used public datasets and remove cells with unclear cell identities to reduce the impact of unknown labels on the fairness of experimental analysis. The details of these datasets is shown in Table 1. They are all available for free download at (https://hemberg-lab.github.io/scRNA.seq.datasets/).

**Table 1:** Real scRNA-seq datasets used in the experiment

No.	Dataset	Cell source	Cell number	Gene number	Cell types
1	Camp1	Human	777	19 020	7
2	Camp2	Human	734	18 927	6
3	Xin	Human	1600	39 851	8
4	Tasic	Mouse	1800	24 058	50
5	Muraro	Human	2122	19 059	9
6	Klein	Mouse	2717	24 175	4
7	Yan	Human	90	20 214	6
8	Zeisel	Mouse	3005	19 972	9
9	Segerstolpe	Human	2166	26 179	12
10	Biase	Mouse	56	25 734	4
11	Treutlein	Mouse	80	23 271	5
12	Goolam	Mouse	124	41 428	5
13	Chen	Mouse	14 437	23 284	47
14	Bhattacherjee	Mouse	24 822	21 000	8

We preprocess the real scRNA-seq data using the Scanpy package [32]. The scRNA-seq data consists of a two-dimensional matrix with cells as rows and genes as columns. For these datasets, we remove genes with expression values of 0 in more than 95$\%$ of cells, normalize and logarithmically transform the data, and then select the top 3000 highly variable genes as input data.

### The proposed scAMAC model

This section provides a detailed introduction to the model structure of scAMAC. The scAMAC model mainly consists of two parts: a denoising deep multi-scale autoencoder and a self-supervised clustering network. The multi-scale autoencoder can be used to obtain the low-dimensional representation and reconstructed data of the raw input data. The self-supervised clustering network utilizes the MSA module to fuse the output results of the autoencoder’s layers, allowing for the integration of information from different layers. This fusion process facilitates the exploration of relationships between cells and mitigates the loss of important data features. Consequently, the fused representation enhances the performance of cell clustering.

Moreover, the network incorporates a self-supervised mechanism that plays a dual role in the training process. On the one hand, it guides the training of the multi-scale autoencoder, enabling the extraction of meaningful features from the input data. On the other hand, it optimizes the overall model by iteratively updating the network parameters based on the self-supervised learning signal.

**Figure 1:**
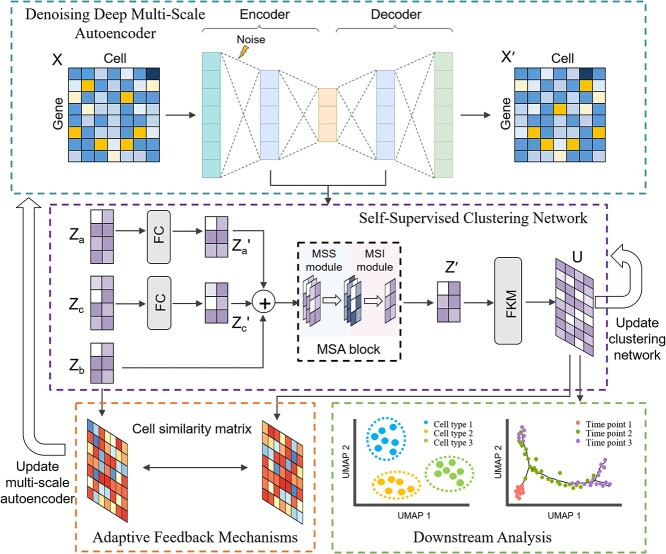
Overall architecture diagram of scAMAC. scAMAC consists of two parts: denoising deep multi-scale autoencoder and self-supervised clustering network. Firstly, the preprocessed gene expression matrix $X$ is fed into the denoising deep multi-scale autoencoder, obtaining the latent feature representation $Z_{b}$ of the hidden layer, as well as the reconstructed data $X^{\prime }$. Then, the outputs of the encoding layer and decoding layer are fed into two fully connected layers to obtain $Z_{a}^{\prime }$ and $Z_{c}^{\prime }$, where the dimensions of $Z_{a}^{\prime }$ and $Z_{c}^{\prime }$ are the same as $Z_{b}$. Finally, $Z_{a}^{\prime}$, $Z_{c}^{\prime}$, and $Z_{b}$ are concatenated and fed into the self-supervised clustering network. The self-supervised clustering network uses the MSA mechanism to capture the relationship between cells and the contribution of each layer of the autoencoder to obtain $Z$. The membership matrix $U$ of the FKM algorithm is calculated based on $Z$ and optimized for the self-supervised clustering network. Meanwhile, $U$ is used to construct a cell similarity matrix to supervise the parameter update of the autoencoder.

As shown in Figure 1, the model takes the gene expression matrix $X$ as input. First, uniform noise is added to the preprocessed data, which is then sent to the autoencoder for training to enhance the robustness of the network. The output of each network layer is $Z_{a}$, $Z_{b}$, and $Z_{c}$. $Z_{a}$ and $Z_{c}$ are transformed into $Z_{a}^{\prime}$ and $Z_{c}^{\prime}$, respectively, through two fully connected layers. $Z_{a}^{\prime}$ and $Z_{c}^{\prime}$ have the same dimensions as $Z_{b}$. Then, $Z_{a}^{\prime}$, $Z_{c}^{\prime}$ and $Z_{b}$ are passed to the self-supervised clustering module. The self-supervised clustering module uses the MSA mechanism to capture the relationship between cells and the contribution of each layer of the autoencoder to obtain $Z$. The membership matrix $U$ is calculated based on $Z$ and optimized by $U$. To implement the self-supervised process within the network, we use the membership matrix $U$ to construct a cell similarity matrix to supervise the parameter updates of the autoencoder.

#### Denoising deep multi-scale autoencoder

For a given scRNA-seq data, its gene expression matrix is represented by $X\in \mathbb{R}^{V\times G}$, where $V$ is the number of cells and $G$ is the dimensionality of genes for each cell. This autoencoder consists of an encoder, a decoder and a hidden layer, which is used to encode and decode gene expression data to obtain the latent features of the data and output the appropriate reconstructed data through the decoder. Specifically, in the encoder, we input the corrupted data $X_{0}$ and obtain the output data $Z_{a}$ in the encoding layer, calculated as follows: 


(1) \begin{align*}& Z_{a}=\phi\left(w_{1} X_{0}+b_{1}\right).\end{align*}

Herein, $\phi $ is the LeakyReLU activation function, $w_{1}$ is the weight matrix of the encoding layer and $b_{1}$ is the bias of the encoding layer. $X_{0} = X + N$, where $N$ is uniform distributed noise. $Z_{a}$ is mapped to $Z_{b}$ via the hidden layer with the following formula: 


(2) \begin{align*}& Z_{b}=\phi\left(w_{2} Z_{a}+b_{2}\right),\end{align*}

where $\phi $ is the LeakyReLU activation function, $w_{2}$ is the weight matrix of the hidden layer and $b_{2}$ is the bias of the hidden layer.

Then, the output data $Z_{c}$ and the reconstructed data $X^{\prime}$ with the same dimension as the encoding layer are obtained through the decoding layer, and the formulas are as follows: 


(3) \begin{align*} & Z_{c} =\phi\left(w_{3} Z_{b}+b_{3}\right), \end{align*}

(4) \begin{align*} & X^{\prime} =\phi\left(w_{4} Z_{c}+b_{4}\right).\end{align*}

In Equations (3) and (4), $\phi $ is the LeakyReLU activation function, $w_{3}$ and $w_{4}$ are the weight matrices of the decoder layer, and $b_{3}$ and $b_{4}$ are the biases of the decoder layer.

In order to enhance the training of the autoencoder and effectively integrate the information between the encoding and decoding layers, we adopt the following loss function to optimize the network: 


(5) \begin{align*}& L_{D_{-} N}=\frac{\sum_{i=1}^{n}\left(X_{i}-X_{i}^{\prime}\right)^{2}}{n}+\frac{\sum_{i=1}^{n}\left(Z_{a_{i}}-Z_{c_{i}}\right)^{2}}{n},\end{align*}

where $n$ represents the number of cells, $X_{i}$ represents the input features of cell $i$, $X_{i}^{\prime }$ represents the reconstructed features of cell $i$, $Z_{a_{i}}$ represents the features extracted by the encoding layer for cell $i$ and $Z_{c_{i}}$ represents the features extracted by the decoding layer for cell $i$.

#### MSA mechanism

The MSA mechanism effectively integrates information from multiple scales and leverages their respective strengths, which consists of two parts: multi-scale synergy (MSS) module and multi-scale integration (MSI) module. In the MSA mechanism, MSS module and MSI module work together to capture spatial information and channel interactions in the input feature map. MSS module is responsible for capturing spatial information and dependencies within channels, while MSI module is responsible for obtaining interactions across channels. The combination of MSS module and MSI module can improve the performance of the model and capture richer feature information.

**Figure 2:**
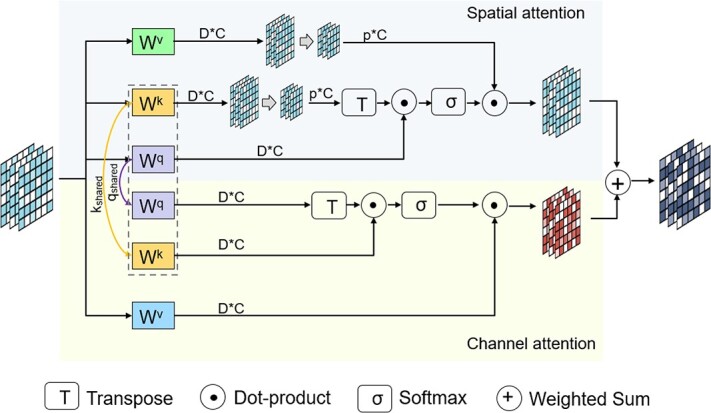
MSS module structural diagram. MSS module consists of spatial attention module and channel attention module. They share the keys-queries weights to generate better and more efficient feature representations.

MSS module consists of spatial attention module and channel attention module, as shown in Figure 2. The spatial attention module is employed to capture the similarity between cells within the same scale, focusing on their spatial relationships. Furthermore, the channel attention module is utilized to explore the deep-level features of cells across different scales. These two modules work in conjunction, sharing the weights of keys and queries, which reduces the parameter count and generates more efficient feature representations.

By incorporating the spatial and channel attention modules, the model can effectively capture both local and global dependencies within the data. The spatial attention module enhances the model’s ability to recognize spatial patterns and capture local correlations between neighboring cells. Meanwhile, the channel attention module allows the model to extract and emphasize the most informative features across different scales, enabling the exploration of deep-level characteristics of cells.

**Figure 3:**
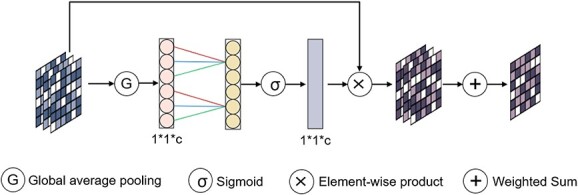
MSI module structural diagram. Given input features, scAMAC applies global average pooling to obtain a summary representation. Subsequently, it utilizes fast 1D convolution to capture inter-channel interaction information. By applying the sigmoid function, it generates channel weights that indicate the importance of each channel. Finally, these weights are used to combine the input feature map in a weighted sum operation.

As illustrated in Figure 3, MSI module performs a non-dimensional reduction local cross-channel interaction strategy, which allows for lightweight capturing of the contributions from different layers of the network. Unlike the channel attention in MSS module, which calculates self-attention on the channel dimension to establish relationships between channels, MSI module uses a global contextual information calculation method to obtain the weight of each channel, thus learning the importance of each network layer in the multi-scale autoencoder.

A regular autoencoder architecture can capture the primary features of cells, but it may overlook the rich structural information contained in each layer of the autoencoder. We further integrate the output results of different network layers within the multi-scale autoencoder to address this issue using the MSA mechanism. This approach aims to explore deeper information between cells by fusing the features at each scale.

To facilitate feature fusion, we first reduce the dimensionality of the decoded layers and their outputs $Z_{a}$ and $Z_{c}$ using fully connected layers. The formula is as follows: 


(6) \begin{align*} & Z_{a}^{\prime}=\phi\left(w_{11} Z_{a}+b_{11}\right), \end{align*}

(7) \begin{align*} & Z_{c}^{\prime}=\phi\left(w_{22} Z_{c}+b_{22}\right).\end{align*}

In Equations (6) and (7), $\phi $ represents the LeakyReLU activation function. $w_{11}$, $w_{22}$, $b_{11}$ and $b_{22}$ are the weight matrix and bias of the fully connected layer network, respectively.

We concatenate $Z_{a}^{\prime }$, $Z_{c}^{\prime }$ and $Z_{b}$, and normalize them: 


(8) \begin{align*}& Z^{\prime}=Z_{a}^{\prime}+Z_{c}^{\prime}+Z_{b}.\end{align*}

Then, we use MSS module to explore the deep features of cells and their interrelationships, with the following formula: 


(9) \begin{align*}& \begin{array}{c} Z_{e} = S A\left(Q_{\text{shared }}, K_{\text{shared }}, V_{\text{spatial }}\right)\\ +C A\left(Q_{\text{shared }}, K_{\text{shared }}, V_{\text{channel }}\right).\\ \text{ s. t. } Q_{\text{shared}} = w_{q} Z^{\prime}+b_{q}, K_{\text{shared}} = w_{k} Z^{\prime}+b_{k}, \\ V_{\text{spatial}} = w_{v s} Z^{\prime}+b_{v s}, V_{\text{channel}} = w_{v c} Z^{\prime}+b_{v c}. \end{array}\end{align*}

here, $SA$ represents the spatial attention module, and $CA$ represents the channel attention module. $Q_{\text{shared}}$, $K_{\text{shared}}$, $V_{\text{spatial}}$ and $V_{\text{channel}}$ are matrices representing shared queries, shared keys, spatial value and channel value vectors, respectively. $w_{q}$, $w_{k}$, $w_{vs}$ and $w_{vc}$ denote the weight matrices of the four different initialized fully connected layers. $b_{q}$, $b_{k}$, $b_{vs}$ and $b_{vc}$ denote the biases of the four different initialized fully connected layers.

Spatial attention module SA is defined as follows: 


(10) \begin{align*}& \begin{array}{c} Z_{s} = \operatorname{softmax}\left(\frac{Q_{\text{shared }} K_{\text{proj }}^{T}}{\sqrt{d_{k}}}\right) \cdot V_{\text{spatial }}^{\prime}. \\ \text{ s. t. } \quad K_{\text{proj}} = w_{p} K_{\text{shared }}+b_{p},\\ V_{\text{spatial}}^{\prime} = w_{s} V_{\text{spatial}}+b_{s}. \end{array}\end{align*}

The weights for spatial projection are denoted as $w_{p}$ and $w_{s}$, and the biases for spatial projection are denoted as $b_{p}$ and $b_{s}$. The dimension of $d_{k}$ is the same as the dimension of the latent feature $Z^{\prime }$, which is used to prevent the softmax values from becoming too large, leading to the partial derivative of the attention mechanism approaching 0.

The formula for channel attention CA is as follows: 


(11) \begin{align*} Z_{c} & = V_{\text{channel }} \cdot \operatorname{softmax}\left(\frac{Q_{\text{shared }}^{T} K_{\text{shared }}}{\sqrt{d_{k}}}\right).\end{align*}

For the output $Z_{e}$ of MSS module, we normalize it again and pass it to MSI module to learn the importance of each network layer in the autoencoder and obtain the final low-dimensional latent feature $Z$.

#### Self-supervised clustering network

We use the low-dimensional latent feature $Z$ to compute the membership matrix $U$ in the FKM algorithm, and optimize the self-supervised clustering network through $U$. Its loss function is as follows: 


(12) \begin{align*}& L_{C_{-} N} = \sum_{i = 1}^{n} \sum_{j = 1}^{k} H_{i j} u_{i j}\left(Z_{i}-C_{j}\right)^{2}.\end{align*}

In Eq. (12), $H_{i j} = \frac{(1+\varepsilon )\left (\sqrt{\left (Z_{i}-C_{j}\right )^{2}}+2 \varepsilon \right )}{\left (\sqrt{\left (Z_{i}-C_{j}\right )^{2}}+\varepsilon \right )^{2}}$ is weight of the loss optimized for self-supervised clustering. $Z_{i}$ represents the latent feature of cell $i$ obtained by the model. $C_{j}$ is the centroid of cluster $j$. $u_{ij}$ is the membership of the $i$th cell in the $j$th cluster. $\varepsilon $ is a balancing factor that affects the robustness of the self-supervised clustering network.

The update formula for cluster center $C_{j}$ is 


(13) \begin{align*}& C_{j} = \frac{\sum_{i = 1}^{n} H_{i j} u_{i j} Z_{i}}{\sum_{i = 1}^{n} H_{i j} u_{i j}}.\end{align*}

The update formula for membership $u_{i j}$ is 


(14) \begin{align*}& u_{i j} = \frac{\exp \left(-\left\|Z_{i}-C_{j}\right\|_{\varepsilon}\right)}{\sum_{j = 1}^{k} \exp \left(-\left\|Z_{i}-C_{j}\right\|_{\varepsilon}\right)},\end{align*}

where $\left \|Z_{i}-C_{j}\right \|_{\varepsilon }=\frac{(1+\varepsilon )\left (Z_{i}-C_{j}\right )^{2}}{\sqrt{\left (Z_{i}-C_{j}\right )^{2}}+\varepsilon }$, $\left \|Z_{i}-C_{j}\right \|_{\varepsilon }$ adaptively adjusts. When $Z_{i}-C_{j}$ is much smaller than $\varepsilon $, $\left \|Z_{i}-C_{j}\right \|_{\varepsilon } \rightarrow \frac{1+\varepsilon }{\varepsilon }\left (Z_{i}-C_{j}\right )^{2}$. When $Z_{i}-C_{j}$ is far greater than $\varepsilon $, $\left \|Z_{i}-C_{j}\right \|_{\varepsilon } \rightarrow (1+\varepsilon ) \sqrt{\left (Z_{i}-C_{j}\right )^{2}} $.

To implement self-supervised learning for the multi-scale autoencoder and incorporate clustering information into the network for improved data reconstruction, we introduce an adaptive feedback mechanism. The corresponding self-supervised loss function is defined as follows: 


(15) \begin{align*}& L_{S} = \frac{\sum_{i, j = 1}^{n}\left(I M_{u_{i}} \star I M_{u_{j}}-I M_{Z_{i}} \star I M_{Z_{j}}\right)^{2}}{n},\end{align*}

where $\star $ denotes the dot product between vectors. ${IM}$ denotes the unit vector. $I M_{Z_{i}} \star I M_{Z_{j}}$ represents the similarity score between different cells.

### Evaluation metrics for clustering

In order to evaluate the clustering performance of scAMAC, two widely used clustering evaluation metrics are used in this paper: Normalized Mutual Information (NMI) [33] and Adjusted Rand Index (ARI) [34]. The larger the values of these metrics, the higher the correspondence between predicted labels and true labels, indicating better clustering performance. We set the true cell labels of scRNA-seq data as $E={E_{1},E_{2},\ldots ,E_{R}}$ and the predicted cell labels as $E^{\prime}={E_{1}^{\prime},E_{2}^{\prime},\ldots ,E_{R}^{\prime}}$.

#### Normalized Mutual Information

NMI is used to measure the similarity between predicted values and true results, ranging from 0 to 1. The formula for calculating NMI is as follows: 


(16) \begin{align*}& N M I=\frac{2 M I\left(E^{\prime}, E\right)}{H\left(E^{\prime}\right)+H(E)}.\end{align*}

$M I\left (E^{\prime }, E\right )$ is used to calculate the mutual information between $E^{\prime }$ and $E$: 


(17) \begin{align*}& M I\left(E^{\prime}, E\right) = \sum_{i = 1}^{R} \sum_{j = 1}^{R} \frac{\left|E_{i}^{\prime} \cap E_{j}\right|}{N} \log \frac{N\left|E_{i}^{\prime} \cap E_{j}\right|}{\left|E^{\prime}\right| \times\left|E_{j}\right|}.\end{align*}

$H\left (E^{\prime }\right ) = -\sum _{i = 1}^{R} \frac{E_{i}^{\prime }}{N} \log \frac{E_{i}^{\prime }}{N}$ and $H(E) = -\sum _{j = 1}^{R} \frac{E_{j}}{N} \log \frac{E_{j}}{N}$ represents the information entropy of labels $L^{\prime }$ and $L$, respectively. $N$ represents the total number of cells.

#### Adjusted Rand Index

ARI is used to measure the overlap between predicted clustering and actual clustering, and its range is [−1, 1]. The formula for ARI is: 


(18) \begin{align*}& A R I = \frac{\sum_{i, j}\left(\begin{array}{c} n_{i j} \\ 2 \end{array}\right)-\frac{\left[\sum_{i}\left(\begin{array}{c} a_{i} \\ 2 \end{array}\right) \sum_{j}\left(\begin{array}{c} b_{j} \\ 2 \end{array}\right)\right]}{\left(\begin{array}{c} n \\ 2 \end{array}\right)}}{\frac{1}{2}\left[\sum_{i}\left(\begin{array}{c} a_{i} \\ 2 \end{array}\right) \sum_{j}\left(\begin{array}{c} b_{j} \\ 2 \end{array}\right)\right]-\frac{\left[\sum_{i}\left(\begin{array}{c} a_{i} \\ 2 \end{array}\right) \sum_{j}\left(\begin{array}{c} b_{j} \\ 2 \end{array}\right)\right]}{\left(\begin{array}{c} n \\ 2 \end{array}\right)}},\end{align*}

$n_{ij}$ represents the number of overlapping cells between $E_{i}^{\prime}$ and $E_{j}$. $a_{i}$ represents the number of cells of type $i$ in $E^{\prime}$, and $b_{j}$ represents the number of cells of type $j$ in $E$.

## RESULTS

### Comparison with other clustering methods

In this section, we comprehensively evaluate the clustering performance of the scAMAC model by applying it to cluster 14 real scRNA-seq datasets and obtaining the final predicted labels. We compare the clustering results of scAMAC with two popular machine learning methods, Seurat and SIMLR, as well as five advanced deep learning methods, including scDeepCluster, Contrastive-sc, scGMAI, scGAC and GraphSCC, all with default parameters. These deep learning methods are based on autoencoders, graph neural networks and contrastive learning. By including a diverse set of clustering techniques, spanning various types of single-cell deep clustering methods, we aim to comprehensively demonstrate the effectiveness of our approach. We use the same preprocessing method to select 3000 highly variable genes from the raw data as input for all methods. Additionally, we use NMI and ARI, two widely recognized clustering metrics, to evaluate the clustering performance of the models. All clustering methods are run 10 times, and we take the average values.

**Figure 4:**
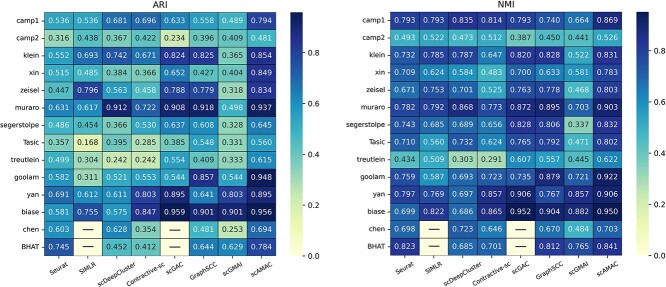
Comparison of clustering metrics between scAMAC and seven other clustering methods. BHAT is short for the Bhattacherjee dataset. SIMLR and scGAC require large memory, so they could not obtain results on the Chen and BHAT datasets.

Figure 4 shows the comparison results of the eight clustering methods on the 14 scRNA-seq datasets. From the figure, we can intuitively see that scAMAC outperforms the other seven deep clustering methods on most of the datasets. Specifically, for the ARI metric, scAMAC achieves the best performance on 11 datasets and ranks second with a very close value to the top on the Biase [35] dataset. For the NMI metric, scAMAC achieves the best performance on 10 datasets and ranks second on the Biase and Chen [36] datasets. All clustering methods perform poorly on the Camp2 [37] and Treutlein [38] datasets, which may be due to the high noise level and small data size of these datasets. Overall, scAMAC still has a significant advantage over other methods.

**Figure 5:**
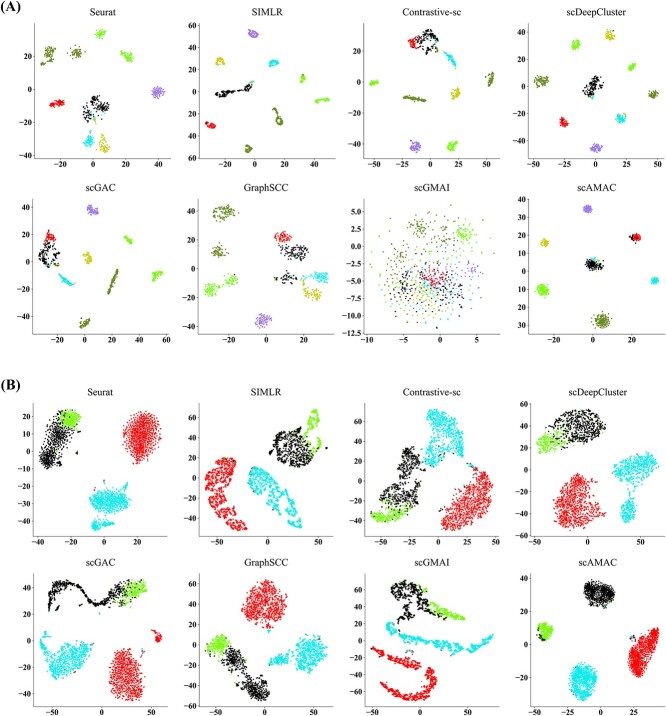
Comparison of two-dimensional visualization of embedded representations. (**A**) Comparison of two-dimensional visualization for different methods on the Camp1 datasets. (**B**) Comparison of two-dimensional visualization for different methods on the Klein datasets.

To obtain a clear biological interpretation of the clustering results, we select two datasets, Camp1 [39] (777 cells) and Klein [40] (2717 cells), which are representative in terms of cell number. For each method, we used t-SNE to visualize the clustering results in 2D space. Figure 5 shows that scAMAC achieves good clustering results on both datasets, with clear boundaries between predicted clusters, better separating different cell types. In contrast, other methods fail to cluster cells with the same label together. For example, in Figure 5(A), Seurat, SIMLR, scDeepCluster, Contrastive-sc, scGAC and GraphSCC tend to divide cells that belong to the same cluster into multiple sub-clusters, while scGMAI mixes multiple cell types together.

**Figure 6:**
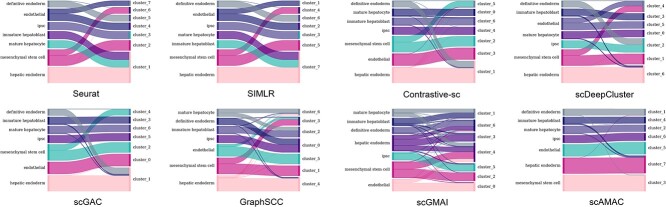
The Sankey diagram comparing the clustering results of scAMAC and seven other methods on the Camp1 dataset.

For the Camp1 dataset, there are seven types of cells, including ‘definitive endoderm’, ‘immature hepatoblast’, ‘ipsc’, ‘hepatic endoderm’, ‘mesenchymal stem cell’, ‘endothelial’ and ‘mature hepatocyte’. Our proposed scAMAC achieves high clustering accuracy on this dataset. To visually compare the performance of clustering methods, we use a Sankey diagram to show the correspondence between the clustering results of each method and the true cell types (Figure 6). It is observed that Seurat and SIMLR methods cluster three large categories of cells into one category, resulting in significant errors. scGMAI and GraphSCC tend to divide cells of the same type into multiple categories, while Contrastive-sc, scDeepCluster and scGAC tend to mix some cells of types with smaller quantities with other cells. In contrast, our proposed method effectively achieves valid division of each type.

### scAMAC is beneficial for recovering gene expression

**Figure 7:**
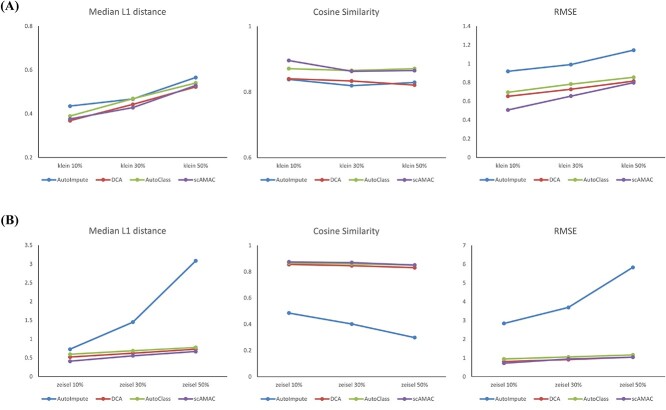
Imputation performance comparison. (**A**) For the Klein dataset with dropout rates of 10, 3 and 50%, the Median L1 distance, RMSE and cosine similarity comparisons were performed between scAMAC and the other three methods. (**B**) For the Zeisel dataset with dropout rates of 10, 30 and 50%, the Median L1 distance, RMSE, and cosine similarity comparisons were performed between scAMAC and the other three methods.

The recovery of gene expression in cells becomes particularly crucial when facing the ‘dropout’ effect in scRNA-seq data. The ‘dropout’ effect refers to the phenomenon in scRNA-seq data where certain genes may have underestimated or entirely missing expression values due to technical limitations and the diversity of cell states. By restoring the gene expression in cells, we can alleviate the dropout effect, enabling a more comprehensive and in-depth understanding of cell states and functions. To evaluate the effectiveness of scAMAC in reconstructing scRNA-seq data, we conduct experiments on two validated cell annotation datasets, Klein and Zeisel [41]. In the experiment, we normalize and logarithmically transform scRNA-seq data using the same preprocessing method. Then, we randomly replace a certain percentage (10, 30 and 50%) of non-zero expression values with zero to simulate the dropout phenomenon. Next, the processed data is reconstructed using AutoImpute, DCA, AutoClass and scAMAC. Finally, we use three evaluation metrics, L1 distance median, RMSE and cosine similarity, as indicators of the ability to recover gene expression. Higher cosine similarity and lower L1 distance median and RMSE indicate better interpolation performance. According to Figure 7, scAMAC is competitive with AutoImpute, DCA and AutoClass, either ranking first or second in all metrics. In fact, when considering all three metrics together, scAMAC performs even better than the other methods. Therefore, scAMAC can effectively alleviate the dropout effect, which is beneficial for the recovery of gene expression.

### scAMAC is beneficial for cell trajectory inference

**Figure 8:**
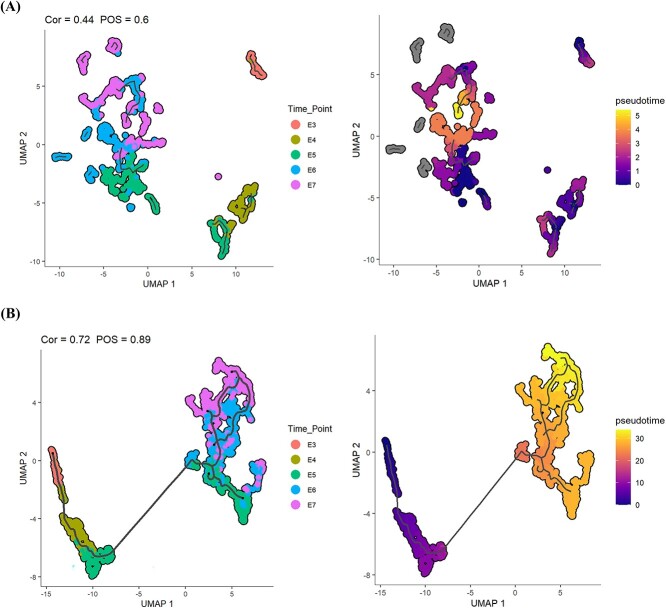
Cell trajectory and pseudo-time plot of the Petropoulos dataset. (**A**) Monocle3 uses raw data as input to reconstruct trajectories and estimate pseudotime. (**B**) Monocle3 uses the low-dimensional representation obtained by scAMAC as input to reconstruct trajectories and estimate pseudotime.

Reconstructing cell trajectories is a common task in scRNA-seq analysis, which is important for studying cell differentiation, cell cycle changes, and cellular responses to external stimuli. Typically, cell trajectory analysis starts by reducing the complexity of gene expression data to select important features more effectively, then constructing the trajectory path of cell dynamic changes, and finally mapping each cell to the corresponding position on this trajectory. Monocle3 [42] is a widely used method for trajectory analysis of scRNA-seq data, which can generate corresponding cell trajectories from the data features of cells. Therefore, in the experiment, we input the low-dimensional cell features obtained by scAMAC and the original data into Monocle3 respectively to obtain cell time trajectories. We also used Pseudo-temporal Ordering Score (POS) and Kendall’s Rank Correlation Score to compare the accuracy of the low-dimensional data representation obtained by scAMAC and the original data in characterizing cell trajectories to demonstrate the effectiveness of data dimensionality reduction by scAMAC. Kendall’s Rank Correlation Score assesses consistency in ordering between two sets of observations, while POS reflects the relationship between predicted pseudo-time order and actual time labels, both aiming for higher scores when alignments occur. We use the common time-series scRNA-seq dataset Petropoulos [43], which consists of scRNA-seq data from embryonic development from day 3 to day 7. From Figure 8, it can be observed that the cell trajectory reconstructed by the original data has a gap with the true time label, and the trajectory is not continuous. In contrast, the cell pseudo-time trajectory inferred by scAMAC has a highly corresponding relationship with the true time label. Furthermore, scAMAC achieved the best POS and Kendall scores, indicating that scAMAC is helpful in reconstructing cell trajectories and can perform effective data dimensionality reduction.

### Collaboration between MSS and MSI module in MSA

**Figure 9:**
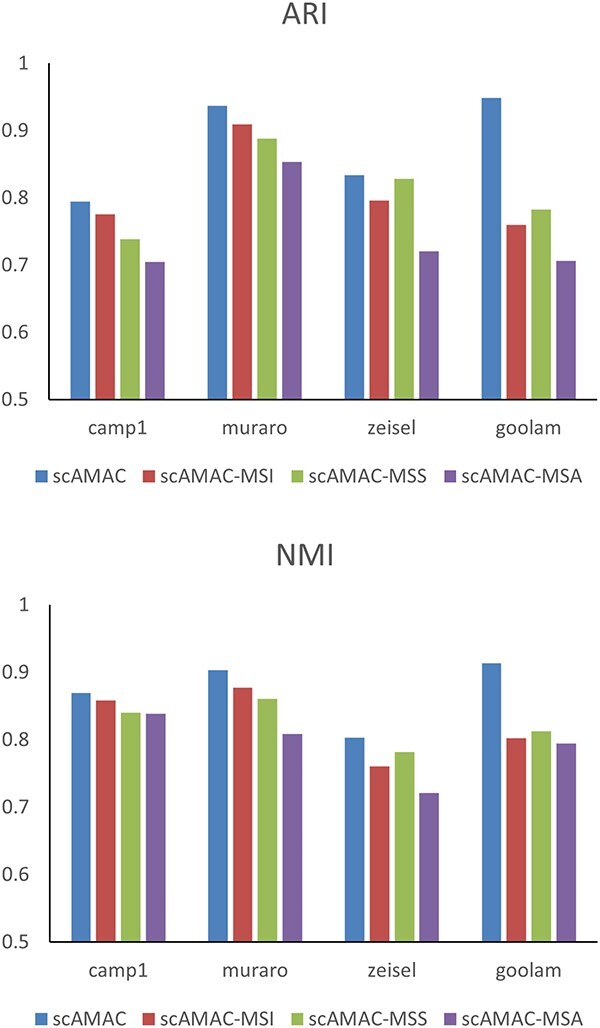
Clustering performance without different attention components in scAMAC.

In this experiment, we explore the collaborative relationship between MSS module and MSI module in MSA mechanism, which are important components of scAMAC, in clustering and data reconstruction. Therefore, we evaluate the necessity of these two components in the model by forming three different models—scAMAC-MSS, scAMAC-MSI and scAMAC-MSA—which represent the removal of MSS module, MSI module and both mechanisms, respectively. We evaluate the average clustering metric values of these models on four datasets: Camp1, Muraro [44], Zeisel and Goolam [45], and the results are shown in Figure 9. We can observe that scAMAC performs the best in both NMI and ARI metrics, followed by scAMAC-MSI and scAMAC-MSS. The scAMAC-MSA model has the worst clustering performance, indicating that both MSS module and MSI module are necessary components of scAMAC for effective clustering performance.

In summary, MSS module and MSI module play important roles in the scAMAC model, and their collaborative relationship further enhances the performance of the model. This indicates that the interaction between MSS module and MSI module has a significant impact on the final result of the model.

### Ablation study

**Figure 10:**
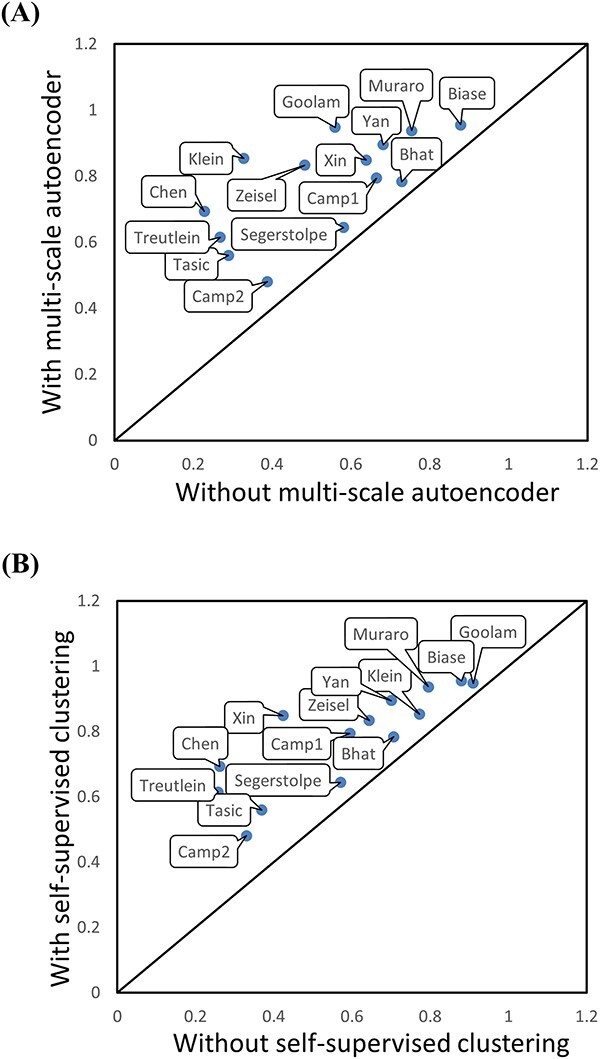
Clustering performance of remove different component in scAMAC. (**A**) Comparison of ARI values with and without using the multi-scale autoencoder in scAMAC. (**B**) Comparison of ARI values with and without using the self-supervised module in scAMAC.

scAMAC consists of two main modules: denoising deep multi-scale autoencoder and self-supervised clustering network. The multi-scale autoencoder integrates feature information from the encoding and decoding layers, while the self-supervised clustering module uncovers deep relationships between cells. Both modules are indispensable parts of the model. We conduct ablation experiments on 14 real datasets to evaluate their impact on clustering results using ARI values. In the experiments, we first reduce the dimensionality of the original data to the same dimensionality as the latent features of the multi-scale autoencoder and use it as input data for the self-supervised clustering module. Then, we compare the results with those obtained using the autoencoder. The comparison results are shown in Figure 10(A). When we remove the multi-scale autoencoder, the clustering performance of all datasets deteriorates, and the ARI values are lower than before. The changes are particularly significant in the Chen and Klein datasets. Next, we directly apply the low-dimensional latent features obtained by the autoencoder to perform common K-means clustering and compare the results with those obtained using the self-supervised clustering module. The comparison results are shown in Figure 10(B). Removing the self-supervised clustering module results in poorer clustering performance, especially on the Xin [46], Chen and Treutlein datasets. In summary, both the multi-scale autoencoder and the self-supervised clustering module play important roles in the model.

## CONCLUSION

This work presents a self-supervised clustering method based on an adaptive multi-scale autoencoder, called scAMAC, which effectively performs cluster assignment and reconstruction of scRNA-seq data. The method utilizes the MSA mechanism to fuse the feature information from the encoder, hidden, and decoder layers of the multi-scale autoencoder, obtaining a more effective representation of latent features of cells. The MSA mechanism allows for the exploration of cell-cell correlations within the same scale and the deep features of cells across different scales. The self-supervised clustering network calculates the membership matrix using the fused latent features and optimizes the clustering network based on the membership matrix. Moreover, the introduction of an adaptive feedback mechanism enables self-supervision of the multi-scale autoencoder through clustering results, facilitating model optimization and ensuring the generation of meaningful reconstructed data.

In simple terms, scAMAC combines information from different layers more effectively, allowing a better understanding of cell relationships. It not only helps cluster cells but also reconstructs data. Compared with existing models, scAMAC stands out by considering these connections more thoroughly, offering a new way to use deep learning for scRNA-seq data analysis and providing fresh insights into understanding cell behaviors. In the experimental section, scAMAC demonstrates its excellent clustering performance on scRNA-seq data from various tissues and scales. It proves to be effective in gene expression recovery and inferring cell trajectories. Overall, scAMAC is a promising method for scRNA-seq data clustering.

**Key Points**

- We develop a novel self-supervised clustering method based on an adaptive multi-scale autoencoder (scAMAC), addressing a crucial oversight in existing deep models. These models often neglect the interconnections among network layers, resulting in the loss of vital structural information within the layers.
- scAMAC stands out by incorporating a Multi-Scale Attention (MSA) mechanism, effectively fusing feature information from the encoder, hidden and decoder layers. This innovative approach enables the exploration of cellular correlations within the same scale while capturing deep features across different scales.
- scAMAC employs an adaptive feedback mechanism, enhancing the representation of cell features. Through experiments, we demonstrate scAMAC’s effectiveness over advanced clustering and imputation methods in both data clustering and reconstruction tasks.

## CODE AVAILABILITY

scAMAC is implemented in Python 3 (version 3.6) using PyTorch (version 1.10.2+cu113). All experiments are conducted on an NVIDIA 3090 GPU with 24 GB of memory. The source codes and supplementary materials are available online at https://github.com/yancy2024/scAMAC.

## Author Biographies

**Dayu Tan** is currently an assistant professor in the Key Laboratory of Intelligent Computing and Signal Processing of Ministry of Education, Institute of Physical Science and Information Technology, Anhui University. His research interests include machine learning, computer vision and data mining.

**Cheng Yang** is a Master’s degree student at the Institute of Physical Science and Information Technology, Anhui University. His research interests include research of bioinformatics and deep learning.

**Jing Wang** is a postdoctoral fellow in the Key Laboratory of Intelligent Computing and Signal Processing of Ministry of Education, School of Artificial Intelligence, Anhui University. Her research interests include research of bioinformatics and machine learning.

**Yansen Su** is a professor at the School of Artificial Intelligence, Anhui University. She is also with Institute of Artificial Intelligence, Hefei Comprehensive National Science Center, 5089 Wangjiang West Road, 230088 Hefei, China. Her research interests include bioinformatics, deep learning and multi-objective optimization.

**Chunhou Zheng** is a professor at the School of Artificial Intelligence, Anhui University. His research interests include machine learning, intelligent information processing and bioinformatics.
